# Isolation, Culture and Characterization of Human Sertoli Cells by Flow Cytometry: Development of Procedure

**Published:** 2017

**Authors:** Mohammad Reza Lakpour, Samaneh Aghajanpour, Morteza Koruji, Abdolhossein Shahverdi, Mohammad Ali Sadighi-Gilani, Marjan Sabbaghian, Reza Aflatoonian, Majid Rajabian-Naghandar

**Affiliations:** 1- Department of Andrology, Reproductive Biomedicine Research Center, Royan Institute for Reproductive Biomedicine, ACECR, Tehran, Iran; 2- Biology Department, Payam Noor University, Tehran, Iran; 3- Department of Endocrinology and Female Infertility, Reproductive Biomedicine Research Center, Royan Institute for Reproductive Biomedicine, ACECR, Tehran, Iran; 4- Department of Anatomical Sciences, School of Medicine, Iran University of Medical Sciences, Tehran, Iran; 5- Cellular and Molecular Research Center, Iran University of Medical Sciences, Tehran, Iran; 6- Department of Embryology, Reproductive Biomedicine Research Center, Royan Institute for Reproductive Biomedicine, ACECR, Tehran, Iran; 7- Department of Urology, Shariati Hospital, Tehran University of Medical Sciences, Tehran, Iran

**Keywords:** Characterization, Culture, Flow cytometry, Isolation, Sertoli cells

## Abstract

**Background:**

The sertoli cells in the testis create unique and safe environment to protect seminiferous tubules from auto antigens and invading pathogens. These cells produce the survival factor of the blood-testis barrier and produce special materials such as androgen binding proteins and contribute to the coordinated action of spermatogenesis. Given that the sertoli cells play an essential role in spermatogenesis and the lack of these cells leads to the disruption of spermatogenesis, it is necessary to investigate the behavior and performance of these cells. To achieve this goal, the cells must first be extracted. The aim of this study was to develop a procedure to isolate, culture, and characterize human sertoli cells.

**Methods:**

In order to isolate the sertoli cells of azoospermia patients who underwent (testicular sperm extraction) TESE surgery, washing up and multi_stage enzyme digestion of single cells, culture on petri dishes impregnated with datura stramonium lectin agglutinin (DSA) were done and then the cells were passaged for several times and isolated. For more purification, flow cytometry method with FSH receptor antibody was used. Immunocytochemistry assays and Elisa test for identification of these cells were employed.

**Results:**

The purification method resulted in more than 97% purity. The nature of sertoli cells was confirmed by morphology evaluation, detecting anti-mullerian hormone in sertoli cell culture media and the presence of FSH receptor on sertoli cells.

**Conclusion:**

This study introduced and applied a method to isolate, culture, and purify human sertoli cells with high purity which made possible further researches on these cells.

## Introduction

The mammalian testis is composed of seminiferous tubules and the interstitial tissue. In addition to the macrophages in the interstitial tissue, interstitium may construct the first line of testicular defense against pathogens from the bloodstream (1). The sertoli cells within the seminiferous tubules play a crucial role to maintain testis as an immune privileged site in which both allo- and auto antigens can be tolerated. These cells also play an important role in spermatogenesis (2).

The sertoli cells are the first cells to differentiate recognizably in the indifferent fetal gonad, an event which enables seminiferous cord formation, prevention of germ cell entry into meiosis and differentiation and function of the leydig cells. Sertoli cells also ensure regression of mullerian ducts via secretion of anti mullerian and inhibin hormones (3). When these events occur, the role of sertoli cell switches during puberty to support spermatogenesis. Without the physical and metabolic support (protection and nourishing) of sertoli cells, germ cell differentiation, meiosis and transformation into spermatozoa would not occur.

Sertoli cells in the basal part-side adjacent cells connected by tight junctions form the blood-testis barrier and seminiferous epithelium are divided into two portions of basal and lumen adjacent that creates a safe environment and protects spermatozoa, keeping it isolated from the blood. Other duties of sertoli cell are phagocytosis of extra proteins of germ cells and preventing the release of sperm (4).

In order to augment our understanding of sertoli cells, it is required to develop isolation approaches of these cells from testis tissues and culture as well as *in vitro* purification.

The most common methods of isolation of sertoli cells and studies in this field were applied in animal models (5–7) and human (8). Nevertheless, the most important multi-step enzymatic digestion was carried out on a number of seminiferous tubules. After using stronger and more efficient enzymes to digest these tubules and cells which mainly contain myoid cells and germ cells, it was cultured on petri dishes coated with DSA lectin. This is a quick method of sertoli cell culture, but one of important disadvantages of this method is that apart from sertoli cells, the other myeloid cells such as fibroblasts attached to the bottom of the petri dish.

This study was conducted to isolate and purify sertoli cells exclusively by FACS sorter in order to understand the unknown and potential roles of these cells.

## Methods

### 

#### Sample collection:

The isolation method chosen was based on previous studies in human and animals. In this study, isolated and cultured sertoli cells from human testes segments were used (with consent of subjects at the beginning of the treatment). These testes biopsies were taken from ten men with obstructive azoospermia (primary infertility) referred to Royan Institute. The average age of men was 30 years. The presence of sperm has been proved in testicular biopsies. All men participated in the study had no history of infection or congenital disorder, and all procedures were approved by Royan ethics committee.

#### Testicular cells isolation:

For isolation of testicular cells, from every ten men with azoospermia, at least 2–4 pieces (3–5 *mm*) of testicular were taken. The samples were divided into small pieces and by shaking and a little pipetting, suspended and washed up with phosphate-buffered saline (PBS; Sigma, USA) containing penicillin- streptomycin and gentamycin and then were placed in dulbecco’s modified eagle medium (DMEM; Gibco, USA) culture media at 37°*C*. They were minced into small pieces and suspended in DMEM, which contained 1 *mg/ml* collagenase, 1 *mg/ml* trypsin, 1 *mg/ml* hyaluronidase and 1 *mg/ml* DNase. All the enzymes were purchased from sigma (St Louis, MO, USA).

After the first enzyme digestion, testicular tissue had been digested to smaller pieces and were pipetted for ten min, then centrifuged at 120 *g* for 5 *min* and resuspended in 3 *ml* of DMEM (repeated three times). During this time, every 10 *min* shaking and pipetting were done for 1 *min* interval. At this stage, most of interstitial, fibroblast and endothelial cells were removed from testis segments. In the next step of digestion, a fresh mixture of DMEM and enzymes to the seminiferous tubules component was added. At this stage, repeated pipetting was done with a pasteur pipette for 4 *min*. The cells were separated from the residual tubule fragments by centrifugation at 542 *g* for 5 *min* at 37°*C*. Then cell suspension was washed twice by fresh medium. The obtained suspension mainly contained sertoli cells, spermatogonial cells and residual interstitial cells.

#### Isolation of sertoli cells:

Sertoli cells were isolated from suspension cells using a modified procedure described by Mirzapour et al. (8) and Scarpino et al. Briefly, at first, about 5 *μg/ml* DSA (Sigma, USA) was dissolved in PBS, then coated dishes were prepared by incubation with DSA at 37°*C* for 60 *min*.

Then, the coated petri dishes were washed with DMEM containing 0.5% bovine serum albumin (BSA; Sigma). After drying the coated dishes, the cell suspension was placed on lectin coated dishes and incubated for 2–3 *hr* at 37°*C*, 5% CO_2_. After incubation, the nonadhering cells were discarded and cells attached to the bottom of the petri dish were sertoli and fibroblast cells which were suspended in DMEM containing 10% fetal bovine serum (FBS, Gibco, UK). The cells were cultured for 3–4 days and every time washed with fresh medium (DMEM with 10% FBS).

Approximately after 4–5 days of the cell confluence, attached sertoli cells were detached by treatment with ethylenediamine tetraacetic acid-trypsin (EDTA-Trypsin) in PBS (Calcium and Magnesium free) (Sigma, USA) for 5 *min* at 37°*C*. This condition causes detachment of cells from bottom of the petri dish. The detached cells were washed again by fresh DMEM with 10% FBS and centrifugation of three times at 645 *g* for 5 *min*. The fresh medium (maximum volume 1 *ml*) was added to supernatant cells. The cells were counted and adjusted to desired densities into each well of 12 well multi dish or culture dishes (3 *cm* culture dishes). This method helped to isolate more cells. The cells viability was evaluated by means of dye exclusion test (0.04% trypan blue solution).

#### Purification of sertoli cells by flow cytometry:

Most of the techniques in other articles for isolating sertoli cells used the lectin. It seems that this procedure was incomplete and involves mesenchymal cells, therefore for further purification, it was decided to isolate the cells with a more specific antibody (Anti FSH receptors). To achieve this purpose, FACS with BD FACS aria II was used.

The cells were detached with 0.02% EDTA/trypsin and washed by PBS plus 1% FBS. For detection of FSH expression on sertoli cells surface, Anti FSH receptors [Rabbit polyclonal antibody to FSH receptor (Abcam, USA)] were used. Then, 20 *μl* from primary antibody FSHr (Abcam, USA) was added to cells in 100 *μl* PBS as a test group. After 45 *min* incubation at 4°*C*, cells were rinsed with 1 *ml* of solution PBS and 1% FBS (centrifuged at 1500 *rpm* for 5 *min* at 4°*C*) and then 300 *μl* of PBS was added to cell sediment. Subsequently, 3 *μl* secondary antibody (Goat polyclonal antibody to rabbit IgG FITC, Abcam, USA) diluted with 1:200 concentration was added to test and control group. Both samples were incubated at a temperature of 4°*C* for 45 *min*, followed by washing with 1 *ml* of solution containing PBS and 1% FBS, centrifuged with 1500 *rpm* for 5 *min* at 4°*C*. The supernatants were removed and sediment has been suspended in 1 *ml* of solution (PBS, EDTA, HEPES buffer, FBS 1%).

Finally, sertoli cells were sorted on the basis of rFSH expression by BD FACS aria II cell sorter, (BD Biosciences).

#### Culture and enrichment of sertoli cells:

Due to low number of obtained sertoli cells after FACS, 12 well multi dishes were used. The sertoli cells obtained from flow cytometry were transferred into one of wells that were rich in culture medium DMEM with 10% FBS. Sertoli cells were incubated at 37°*C* and 5% of CO_2_ (9) and their medium was replaced each 48 *hr*. Cultivation period was 2 weeks.

In order to improve the conditions of FSH, sertoli cells with a concentration of 7000 *IU/mg* were added to the wells.

Human FSH (follicular stimulating hormone) hormone is produced by Sigma Company. Three to 5 days after incubation of sertoli cells, these cells formed a confluent layer. Viability and count of sertoli cells were evaluated by 0.04% trypan blue solution.

### Characterization of sertoli cells

#### Immunocytochemistry:

The sertoli cells cultured on a well of a 12-well multi dish were evaluated with FSH receptors as a marker. These cells were fixed with 4% formaldehyde for 2 *min* and also permeabilized with 0.3% triton X-100 in PBS for 15 *min* at room temperature. The blocking was performed by incubation with 10% normal goat serum in PBS at room temperature for 30 *min*. The cells were incubated with the Rabbit polyclonal anti FSH receptor antibody (Abcam, USA) diluted by 1/200 concentration for 24 *hr* at 4°*C*. The cells were incubated with the secondary anti body (Goat FITC-conjugated anti Rabbit IgG) (Abcam, USA) with 1/100 concentration. Finally, cells were mounted with mounting solution (Vector Laboratories Inc., Burlingame, CA) and then examined under a inverted fluorescence microscope (IX-71; Olympus).

#### ELISA:

Anti mullerian hormone (AMH) secreted by sertoli cells in the culture medium was measured by ELISA kits (Anshlab, Germany) according to manufacturer’s instructions.

## Results

### 

#### Morphological study:

**Figure 1.**
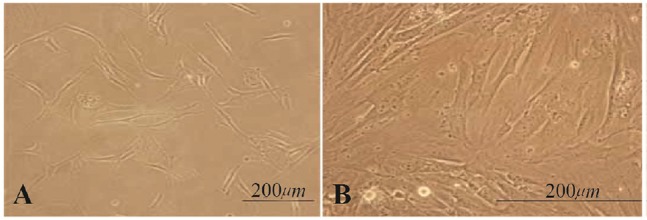
A: Sertoli cells 4–5 days after culture with extended expansions; B: the isolated and proliferated cell population created a monolayer of cells after 2 weeks of cultivation

The sertoli cells were evidenced by their nuclear morphology. These cells had an irregular outline with a granular appearance. In general, the sertoli cells lost their long cytoplasmic extensions after enzymatic digestion and were transformed into round cells with crenated edges (10). After 1–2 weeks of culture, they produced extensions, were flattened and attempted to make contact with other cells. After several passages, these cells made monolayer cells at the bottom of petri dish (Figure 1).

#### Investigating FSH receptors on the surface of the sertoli cells:

**Figure 2.**
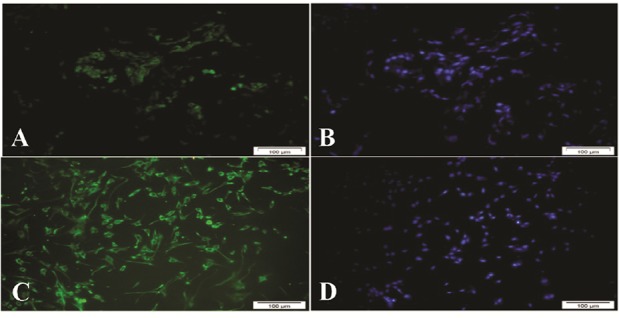
A, B: FSH receptor in human testicular tissue as a positive control: C, D: FSH receptor in cultured sertoli cells

Since FSH receptors exist on sertoli cells, this receptor was evaluated as a sertoli cell-specific marker. Immunocytochemistry staining of sertoli cells showed that the FSH receptor is expressed on the surface of sertoli cells. In this study, the human testis tissue was used as a positive control (Figure 2).

#### Purification of the sertoli cells:

Total count of sertoli cells at the first week after culture were 1 × 10^6^/*ml*. These cells were isolated by FACS sorter. These cells that have rFSH attached to antibodies are detected by the device. Percentage positive sertoli cells for rFSH after flow cytometry were 25%. Finally sertoli cells with a purity of 97% ± 1.26 were sorted.

## Discussion

For several reasons, sertoli cells are important in infertility of men:

1. These cells can be used in the research for cell proliferation and mitotic divisions in extracellular environment (6).
2. In order to improve the spermatogenesis, sertoli cells can be frozen and then they can be transplanted into the testes of infertile men.
3. They can be used in investigating the existence and gene expression of various receptors on sertoli cells such as androgenic hormones and TLR receptors.
4. The sertoli cells can be used as a feeder layer for culturing human testicular cells from patients with maturation arrest in spermatogenesis in control and experimental groups (8).

In this study, a new method for the isolation and purification of the human sertoli cells of testis tissue was developed. This method is very accurate and rapid. Several methods for the isolation of sertoli cells have been studied in human (11) and animals (6), each of which has advantages and limitations, but our method is quick and accurate. In previous studies with common procedures, it seems that sertoli cells were not isolated. Scarpino et al.’s and other studies (6, 8, 11) were based on cells attaching to petri dish by DSA; however, other cells such as fibroblasts cells were attached to the Petri dish. It is thought that the only sertoli cells attached to DSA and their identifications were based on only vimentin antibody, while DSA and vimentin antibody are not specific merely to sertoli cells and vimentin antibody gets positive for all mesenchymal cells such as fibroblasts and sertoli cells. Therefore, the isolated cells were mixed and as a result, they thought that most of cells were sertoli cells, but they were not.

Another new method was recruited in this study as well. Human FSH was used in sertoli cells culture medium. The used human FSH reduces duration of cell culture and allows achieving pure cells in less time. FSH affects the performance and further the growth of sertoli cells (12).

In this study, previous methods were modified and sertoli cells with high purity were obtained. The extracted cells were subjected to the most accurate methods for evaluation and monitoring, in so far as the nature of these cells as the sertoli cells was proven. Methods such as flow cytometry, immunocystochemical and Elisa were used and confirmed.

## Conclusion

Up to now, several methods are used to isolate the sertoli cells that all had advantages and limitations. All of these methods were based on attaching cells to the petri dish impregnated with DSA lectin. This process lets sertoli cells attach to the bottom of the dish in a mono layer but in this process, the other mesenchymal cells such as fibroblasts are also attached to the bottom of the dish. So, there would not be a pure population of sertoli cells and these cells need to be isolated from other cells and then purified. In this study, sertoli cells isolated by the FACS sorter had more than 97% purity and it facilitates the future research on these cells and therapeutic role of them through cell transplantation can be better studied.
